# Cardiology's best friend: Using naturally occurring disease in dogs to understand heart disease in humans

**DOI:** 10.1016/j.jmccpl.2025.100474

**Published:** 2025-07-04

**Authors:** W. Glen Pyle

**Affiliations:** IMPART Team Canada Investigator Network, Dalhousie Medicine, Saint John, NB, Canada; Women's Health Research Institute at BC Women's Hospital + Health Centre, Vancouver, BC, Canada

**Keywords:** Canine, Comparative, Dilated cardiomyopathy, Arrhythmogenic right ventricular cardiomyopathy, Mitral valve disease

## Abstract

Heart diseases are a leading cause of death globally. Laboratory and preclinical animal models of disease have been critical in advancing our understanding of the mechanisms of pathology, creating diagnostic tools, and developing therapeutic interventions. However, fundamental biological dissimilarities between humans and rodents limits their usefulness in research, and the induction of disease in an otherwise healthy animal creates unrealistic conditions under which diseases are typically studied. Dogs are at high risk of acquiring and dying from several naturally occurring heart disorders that also affect people. The spontaneous nature of these conditions, along with highly similar cardiovascular systems, offers unique opportunities to investigate cardiovascular disease in a more relevant model for humans. This review focuses on three common cardiac conditions that impact humans and dogs: dilated cardiomyopathy, arrhythmogenic right ventricular cardiomyopathy, and mitral valve disease – comparing mechanisms of disease, diagnostics, and treatments, to identify strengths and present limitations of their utility. It is noted that the benefits of this research are bidirectional, with the potential to translate knowledge and clinical tools used in veterinary medicine to human patients, and vice versa.

## Graphical abstract

**Figure:**
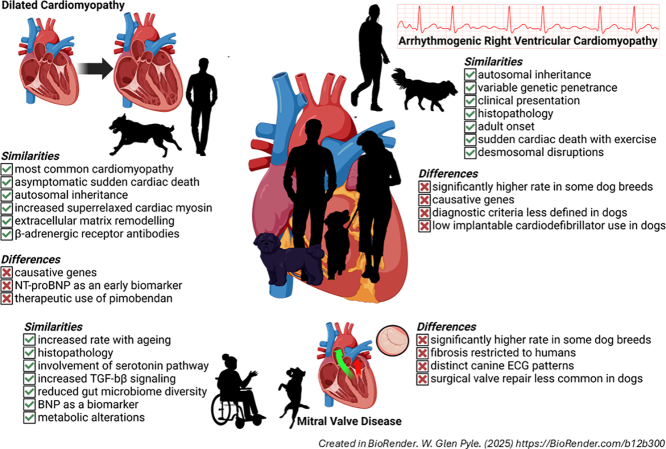

## 1 Introduction

Modeling heart conditions in laboratory animals is critical to gain insight into the biological mechanisms of disease and for the development of therapies. Without question, research done using rodents and other common laboratory models has yielded discoveries that have positively impacted human health. Despite the advances made with rodents, they inherently possess characteristics that limit their utility for translation to human conditions, demanding subsequent investigation with pigs, sheep, and primates, whose cardiovascular systems are more similar to humans. But even these models have significant limitations, not the least of which is that disease is typically induced in an otherwise healthy animal, creating an incredulous timeline and unrealistic context for disease development.

Dogs possess a cardiovascular system that is comparable to humans, allowing them to be used in fundamental research. In general, cardiovascular anatomy is similar between dogs and humans, including a four-chamber heart with flow controlled by valves that are structurally and functionally comparable [1]. Resting heart rates of 60 to 100 bpm are reported in humans and dogs, although smaller breed dogs tend to have resting hearts rates that are in the range of 120 to 160 bpm. Mechanisms to regulate cardiac contractility are also alike between the species, with cardiac myocytes demonstrating similar responses to pharmacological agents [2]. The electrical conducting systems are anatomically similar between humans and dogs, and studies find similar ion channel contributions [3,4], although inward rectifier potassium currents in cardiomyocytes are significantly larger in dogs [4]. Cardiovascular differences between dogs and humans include an ovoid shape of the canine heart with a blunt apex that tilts more ventrally than humans [1]; the predominance of the left circumflex artery over the left anterior descending artery, a greater number of afferent veins in the coronary circulation, and a robust coronary collateral circulation [5,6]; and smaller but prolonged cardiomyocyte calcium transients [2]. However, even in studies that highlight cardiovascular differences between humans and dogs, the general conclusions are that inter-species similarities outweigh the differences, and that dogs are good models for human cardiovascular physiology and pathophysiology [2,4]. Among the conditions for which canine models have proven to be useful in advancing our understanding of disease are acute myocardial infarction, tachycardic heart failure, and atherosclerosis [7,8] and the development of therapies including stem cells [9] and CRISPR genome editing [10].

As with other so-called “large” animal models, the induction of otherwise artificial disease in research dogs is a significant limitation to recapitulating the human disease condition. However, unbeknownst to a considerable section of the research community outside of veterinary medicine, several breeds of dog have naturally occurring heart conditions that closely match their human equivalencies. For example, canine dilated cardiomyopathy (DCM) was first described in the scientific literature in a 1970 abstract [11], although earlier work by Luginbühl and Detweiler [12] made reference to the condition. Together these studies represent the first published reports on canine DCM.

DCM is so prevalent in Doberman Pinschers that regular screening for DCM is recommended for this breed [13]. Despite the long history and high frequency of DCM in Dobermans, a 1990 study “found” a significant number of apparently health Dobermans with DCM and warned against using the breed to “study normal cardiac physiology” [14]. This oversight of a well-established form of heart failure in dogs underscores the need for closer connections between veterinary and human medicine.

The spontaneous development of cardiac pathology in dogs permits the investigation of disease; analysis of diagnostic testing; and trialling of therapies in a more relevant context than induced animal models, as well as reducing animal use by studying animals that naturally develop disease. Life expectancy in dogs is a fraction of that of humans which means that the timeframe, while natural, is shorter, allowing for a more efficient and faster investigation of disease mechanisms and treatments. The combination of spontaneous disease occurrence, compressed timeline, and a similar cardiovascular system makes dogs an ideal model to study cardiac conditions; identify pathological mechanisms; and test novel therapies and diagnostic tools in the clinical environment of veterinary medicine.

The purpose of this review is to present some naturally occurring cardiac diseases that commonly develop in dogs – including the use of novel diagnostic tools and therapies that may be translatable to humans – to illustrate the potential to draw from research using these animals and apply knowledge to human conditions (Table 1).

**Table 1:** Comparison of cardiac conditions commonly found in dogs and humans.

Condition	Species	Aetiology/mechanisms	Diagnostics	Interventions
DCM	Human	• >60 identified genes • Sarcomeric mutations • Enteroviruses • Pregnancy	• CMR	• LVAD • Transplantation • SGLT 2 inhibitors • ARB/neprilysin inhibitors • MRA • β-blockers
	Shared	• Autosomal dominant • Titin, RNF207, PRKAA2, RPM20 mutations • Viral myocarditis • Toxicity • Superrelaxed myosin	• Family history • ECG • BNP • Troponin • Echocardiography	• Diuretics • ACE inhibitors
	Canine	• PDK4 • Parvoviruses		• Anti-arrhythmics • Pimobendan
ARVC	Human	• 16 genes	• Ventricular structure-function	• Anti-arrhythmics • ICD
	Shared	• Autosominal dominant • Fibro-fatty replacement • Desmosomal disruptions • Adult onset	• Cardiac arrhythmias • Family history	• β-Blockers
	Canine	• Striatin mutation		
MMVD	Human	• Fibrosis • Distinct phenotypes	• Transesophageal echocardiography • CMR	• Valve repair/replacement
	Shared	• Leaflet thickening • Activated myofibroblasts • Disrupted ECM • Age-dependent • ↑Serotonin • ↑TGF-β • ↓Gutmicrobiome diversity		• Vasodilators
	Canine	• Homogenous phenotype	• Transthoracic echocardiography	• ACE inhibitors • Pimobendan • Sodium restriction

Characteristics unique to each species are listed in their respective rows, with shared elements in the row in between. *Abbreviations*: DCM, dilated cardiomyopathy; ARVC, arrhythmogenic right ventricular cardiomyopathy; MMVD, Myxomatous mitral valve degeneration; CMR, Cardiac magnetic resonance; BNP, B-type natriuretic peptide; LVAD, left ventricular assist device; SGLT2, Sodium-glucose co-transporter 2; ARB, angiotensin receptor blockers; ACE, angiotensin-converting enzyme; MRA, mineralocorticoid receptor antagonists; ICD, implantable cardiodefibrillators; ECM, extracellular matrix.

## 2 Genetic aetiologies of heart disease

### 2.1 DCM

DCM is a leading cause of heart failure in humans [15], although its true prevalence is hampered by a lack of population level epidemiological data [16]. Approximately 30–50 % of DCM cases in humans are familial [17,18] with over 60 genes proposed to be causative [19]. Despite the perception of DCM as a condition linked to specific genes, many cases are polygenic or require triggering conditions such as infection, pregnancy, or alcohol use to allow expression of the disease phenotype [16]. People who develop DCM may be largely asymptomatic for many years, although electrocardiographic (ECG) and echocardiographic screening can identify early signs of the disease [16].

Canine DCM has similar familial, autosomal inheritance patterns to humans [14,[20], [21], [22]], and comparably high rates of occurrence: in dogs, DCM is the most common cardiomyopathy, and second most common cardiac condition [23]. DCM is the most common cardiac disease in large and giant breed dogs [[24], [25], [26]]. Among the dog breeds that are more commonly affected by DCM are Great Danes, Doberman Pinschers, Portuguese Water Dogs, Irish Wolfhounds, Newfoundlands, Boxers, Welsh Springer Spaniels, Cocker Spaniels, Airedale terriers, St. Bernards, Standard poodles, Scottish Deerhounds, and Afghan Hounds [25,27,28]. Doberman Pinschers are particularly at risk for DCM with a prevalence ranging from 45 to 63 % [[29], [30], [31]].

In Doberman Pinschers, DCM is an autosomal dominant condition with two distinct histological forms: a fatty infiltrative-degenerative type, and an attenuated wavy fibre form [32]. DCM manifests in two stages in Doberman Pinschers: the occult stage and congestive heart failure. Similar to humans, the occult stage has no overt signs that are detectable by owners, but ECG changes including premature ventricular contractions [31] and atrial fibrillation [25] can be detected with screening, and biomarkers such as N-terminal pro-brain natriuretic peptide (NT-proBNP) may increase [31]. Regular screening for Doberman Pinschers including Holter monitors are recommended, as treatments started in the occult phase delay the onset of heart failure [33]. The spiralling decline from diagnosis to death is particularly rapid in Doberman Pinschers, averaging 8 weeks in the absence of effective treatments [31].

Despite the lack of clear clinical symptoms, the occult phase represents a significant threat for Doberman Pinschers: one-third of dogs with DCM die of sudden cardiac arrest in this phase [29,[34], [35], [36]], a rate similar to human populations [37]. In addition to sudden cardiac arrest, human and canine DCM share several other key symptoms including syncope, exercise intolerance, cardiac arrhythmias, and pulmonary congestion [38,39].

### 2.2 Arrhythmogenic right ventricular cardiomyopathy (ARVC)

ARVC is not an arrhythmic-based condition from the perspective that arrhythmias cause ARVC, but rather the cardiac changes associated with ARVC produce characteristic arrhythmias. The condition was originally described in humans by Fontaine and colleagues [40] and in dogs by Harpster [41] around the same time, although the descriptions are remarkably similar to a “heart being overload with fat” noted by Laennec in 1819, and a familial heart condition identified by Lancisi in 1736 [42].

ARVC is a familial cardiomyopathy that is inherited in an autosomal dominant manner with variable penetrance in both humans and dogs [43,44]. The condition is characterized by a fibro-fatty replacement of the right ventricular myocardium that disrupts both electrical and mechanical function, leading to an increased risk of ventricular arrhythmias, reduced contractile function, and sudden cardiac death [45,46]. Historically ARVC was identified as a condition affecting the right ventricle, but more recently biventricular and even primarily left ventricular involvement has been described. This has led to the proposal that the condition be called “arrhythmogenic cardiomyopathy” [47] although ARVC continues to be more commonly used. In people, the prevalence of ARVC is between 1 in 2000–5000, with a slight bias towards males [47]. In dogs, the overall rate is similar, except in Boxers which have rates that are typically quoted as ∼25 % [28]. English and American Bulldogs also have elevated rates of ARVC, albeit lower than those seen in Boxers. The form of ARVC that afflicts Bulldogs appears to be more severe than that of Boxers [48].

ARVC in the Boxer is extraordinarily similar to the human condition in terms of its clinical presentation; pathological timeline; histopathological characteristics; and outcomes [46,47,49]. ARVC is marked by ventricular tachyarrhythmias originating from the right side of the heart; syncopal episodes; congestive heart failure; and sudden cardiac death in both humans and dogs. Right ventricular mechanical dysfunction in dogs is similar to the changes observed in people [49,50]. Most dogs do not develop overt symptoms of ARVC until middle-age [51] which fits with the more frequent adult-onset reported in people [47,49]. Interestingly, fatty infiltrates are not readily visible in young dogs, but become more common with age and disease progression [50,52]. Finally, a common feature of human and canine ARVC is the exacerbation of the disease by training [51] and the association between sudden cardiac death and exercise [47,49].

## 3 Non-genetic aetiologies of heart disease

### 3.1 Mitral valve disease

Myxomatous mitral valve degeneration (MMVD) is the leading indication for valve replacement or repair in humans [53], and is the most prevalent acquired heart disease among dogs, accounting for 75 % of all canine heart disease [54]. The global estimates of MMVD in humans is 2-3 % [55], with the highest prevalence emerging with ageing such that 10 % of people have mitral valve insufficiency by age 65 [56]. Dogs also demonstrate an age-dependent rate of MMVD, but with rates much higher than humans in certain breeds. In medium and small breed dogs (broadly defined as a body weight < 15 kg) such as Miniature Poodles, Miniature Schnauzers, Chihuahuas, Pomeranians, Fox Terriers, Cocker Spaniels, and Pekingese breeds, MMVD afflicts 30–70 % of dogs, with some breeds like the Cavalier King Charles Spaniel experiencing an almost 100 % prevalence of the disease [54,57]. MMVD shows significant clinical variabilities depending on breed [58], and while valve disease is more common among small breed dogs, disease progression is more rapid in larger dogs [59].

The histological changes and clinical development of MMVD are well characterized, but the aetiology and molecular basis of disease remain poorly understood. The gross structural features of the mitral valve such as the architecture of the annulus; continuity of the anterior leaflet with the aortic cusps; and branching network of the chordae tendonae, are similar between dogs and humans, offering a comparable physiological model of the mitral valve [60]. In general, MMVD is marked by leaflet thickening, activated myofibroblasts, and a disrupted extracellular matrix [61]. One key difference between humans and dogs is that only the human condition is marked with fibrosis [62]. Nonetheless, the generally similar histological and structural changes of the mitral valve – including extracellular build-up of proteoglycans and disruptions in the extracellular matrix of the leaflets – advances opportunities for comparative investigations of disease and makes the dog the best model to study the human condition [60,61].

### 3.2 DCM

In addition to a familial basis, DCM has a number of non-genetic aetiologies including infection, toxicity, ischaemia, nutritional deficiencies, and endocrine disorders [63]. Myocarditis is a common non-genetic cause of DCM in both humans and dogs [64,65]. Parovovirus is the most common pathogen detected in dogs with myocarditis [66]. In humans, parvovirus is also cited as the most common viral pathogen found in myocarditis cases [67], although the role of parovovirus in human cases is less clear: high positivity rates have been reported in human cases, but similarly high rates are found in myocarditis-negative people [68,69]. Enteroviruses are also frequently linked to viral myocarditis in humans, with Coxsackievirus B3 being the most common subtype [70]. Regardless of the cause, myocarditis is histopathologically similar in human and dog cases, marked by an inflammatory infiltrate that may or may not be associated with necrosis. Another common feature between canine and human cases of myocarditis is a bias towards young adults or juveniles [66], although a disproportionate number of cases in males is only reported in human studies [66]. In general, the similarities in canine and human myocarditis offer the possibility to collect comparative data on pathogenesis, diagnosis, and treatment, and translate this information to human and veterinary medicine.

The laboratory animal model for human myocarditis research is mice infected with Coxsackie B virus, but there are no animal models for parvovirus-associated myocarditis [67]. The naturally occurring parvovirus infections that are common among canine patients offers the opportunity to study this form of myocarditis in a more comparable cardiovascular system to humans, and under temporal conditions that more closely resemble human cases. Recent work by Di Loria and colleagues [71] used next-generation sequencing to identify gene expression changes in dogs with viral myocarditis and found their results were comparable to human studies, supporting the validity of cross-species comparisons in myocarditis [66]. This comparative research is particularly important given the number of viral infections in people that do not lead to post-myocarditis DCM [72,73]. These cases suggest genetic-immune-environmental interactions are critical to pathogenesis [69,73], although what these factors are remains largely unknown. A review by Tschope et al. [69] highlighted knowledge gaps with myocarditis in humans and noted the need for experimental models that better model the human systems involved in myocarditis, a request that is well matched by canine patients. Studies looking at human and canine cases of parvovirus infections could be subjected to sequencing analysis as a step towards identifying genetic variants more commonly associated with DCM, and blood work and post-mortem or biopsy analysis of myocardium could shed light on the pathogenic mechanisms responsible for the increased susceptibility to post-myocarditis DCM. One significant limitation of using veterinary patients as models of viral myocarditis is that while most dogs are vaccinated against parvovirus, owners who do not follow recommended vaccination schedules are less trusting of veterinarians [74]. This fractured relationship means that the dogs most likely to be infected (i.e. unvaccinated dogs) are less likely to been seen for care, which in turn limits access to naturally occurring cases of viral myocarditis.

Hypothyroidism is a relatively common condition in humans, but it rarely causes DCM because of robust screening programs and effective treatments [75]. Nonetheless, even subclinical hypothyroidism in people increases the risk of cardiovascular mortality by up to 80 % [76]. Similarly, one of the most common endocrine disorders in dogs is hypothyroidism. Dobermans have an unusually high rate of hypothyroidism compared to other breeds [[77], [78], [79], [80]]. Although the risk of DCM in Doberman Pinschers with hypothyroidism increases 1.76-fold, it does not appear to have a driving role in the development of DCM [81]. Despite the inability to identify hypothyroidism as a common cause of DCM, the relatively high frequency of the condition in both humans and dogs provides an opportunity to investigate cardiovascular changes that do develop in Doberman Pinschers and people to generate insight into the broad cardiovascular disease risks associated with thyroid disruption.

## 4 Mechanisms of heart disease

### 4.1 DCM

Most DCM cases in Doberman Pinschers are hereditary. Despite the relative commonality of this form of cardiomyopathy, little is known about its genetic basis, with only a handful of mutations identified [23]. Similarly, human cases are largely genetically based; but in contrast to the canine condition, over 60 genes have been linked to DCM [19]. Interestingly, the known mutations in people only account for 20-35 % of familial cases [82]. Recent studies have begun to posit the idea that many cases of DCM are complex polygenic conditions brought about by a combination of genetic variants and environmental factors that allow manifestation of the disease [19,82]. Together these findings underscore the lack of knowledge of the genetic basis for DCM in both species, and the need for further investigation.

The large number of human and canine DCM cases that remain unexplained, coupled with the similarities in pathogenesis between canine and human patients, could benefit from comparative investigations. Genetic and epigenetic analysis of samples taken from dogs, combined with pedigree analysis, has been used to identify or rule out putative drivers of disease [22,83]. Post-mortem myocardial samples from canine patients have been used in fundamental science investigations designed to identify molecular mechanisms downstream of mutations [84,85] and test novel therapies [85]. The simultaneous investigation of human samples has been critical to identify common and unique elements of DCM in each species [22,84,86].

A review and comparison of some causes in canine and human DCM lays the foundation for further pursuits by presenting what is known and what remains to be answered.

#### 4.1.1 PDK4

A genome-wide association study of Doberman Pinschers with DCM by Meurs and colleagues [87] reported a 16-base pair deletion in intron 10 of the pyruvate dehydrogenase kinase 4 (PDK4) gene. The mutation is predicted to produce a less activate form of PDK4 and shift energy supply from fatty acid oxidation towards a greater reliance on glucose oxidation [87]. Expression of the mutant form of PDK4 in canine dermal fibroblasts revealed putative pathological changes including an impaired mitochondrial response to starvation stress and an increased rate of cellular apoptosis, effects that were reversed with adeno-associated virus mediated delivery of wildtype PDK4 [88,89]. Studies using Doberman Pinschers with DCM were unable to directly measure PDK activity to confirm the hypothetical connection with DCM, and subsequent studies found that the PDK4 deletion was only expressed in 16 % of Doberman Pinschers with DCM, with similar levels in healthy dogs [90].

While the role for a less active form of PDK4 in canine DCM remains under investigation, there is translational potential for this model in human cardiology. Reduced glucose oxidation through increased PDK4 activity has been hypothesized to be involved in human heart failure associated with obesity and diabetes [91]. Studies have supported this model by showing that PDK4 deletion prevents cardiac dysfunction and normalizes glucose oxidation in the face of angiotensin II-induced hypertrophy [92]. Based on these findings, PDK4 inhibitors have been produced as putative treatments for heart failure with reduced ejection fraction [93]. The paradoxical findings of a pathological association with PDK4 inhibition in dogs and the therapeutic potential for PDK4 inhibitors present a significant question about the use of PDK4 inhibitors for human patients. It also suggests that the use of dichloroacetate (whose mechanism is proposed to involve PDK4 inhibition) to treat heart failure patients [94] may work by alternate means, including activation of the pentose phosphate pathway as an alternate energy source [93]. Research using Doberman Pinschers with the PDK4 mutation identified by Meurs and colleagues offers the potential to further explore and investigate the therapeutic strategy of PDK4 inhibition in a more relevant animal model than the rodent models used thus far.

#### 4.1.2 Titin

Meurs and colleagues also identified a pathological missense mutation in an Ig-like domain of the I-band of titin [95]. Unlike titin mutations in human DCM, titin expression was not impacted nor was passive tension, with only a small effect on active tension generation. Our targeted investigation of genes that are commonly affected in human DCM did not detect a titin mutation, nor did it reveal other potentially causative mutations for Doberman Pinschers, although several single nucleotide polymorphisms were identified [83].

Titin truncating variants are commonly associated with DCM, but less is known about I-band variants like the one reported by Meurs et al. [95]. In an investigation of 15 human DCM patients, Mukhopadhyay et al. [96] identified 88 exonic variants and found that the number of I-band variants connected to DCM was exceeded only by those in the A-band region. Of the I-band variants identified, 37.5 % were labeled as “damaging” or “deleterious”, while 44.3 % of A-band variants similarly labeled. Mutations in the PEVK rich spring-like domain of the I-band region can alter titin stiffness and cellular passive tension, but this region also contains two major signaling hubs that influence cardiac function [97]. Of particular interest are members of the four and a half LIM (FHL) family and the Cardiac Ankyrin Repeat Protein (CARP) which dock in the I-band, and mediate critical signaling events that have been linked to myocardial diseases, including DCM [[98], [99], [100]]. The uncoupling between passive stiffness changes and potentially disrupted intracellular signaling that may underlie DCM in the Doberman Pinscher warrants further investigation as a putative cause. Moreover, increasing awareness of titin I-band variants in humans and their connection to cardiac disease makes this an attractive area to target with novel therapies [97], and Doberman Pinscher DCM patients could serve as a valuable model in which to test these interventions.

#### 4.1.3 Polygenic causes

Recently Niskanen and colleagues [22] identified novel mutations in RNF207 and PRKAA2 that are shared by human and canine DCM patients. The realization that individual mutations have weaker prediction values has led some to advance a multi-locus cause for DCM in Dobermans [27], and more recently in humans [101]. This evolution of thought towards a potentially polygenic basis of DCM in humans and dogs mirrors a similar progression seen in human hypertrophic cardiomyopathy research, to the point where the majority of hypertrophic cardiomyopathy cases are believed to be the product of complex interactions of incomplete penetrance and variable expression of disease variants [102]. The polygenic changes detected in Doberman Pinschers with DCM could be used to explore the interactive model of heart failure in a context that mirrors the human disease, both in terms of investigating disease mechanisms and developing new diagnostic and therapeutic tools.

#### 4.1.4 Calcium removal

Doberman Pinschers have the highest rate of DCM among all breeds of dogs, but they are not alone in being afflicted with high rates of familial DCM. Welsh Springer Spaniels have a missense mutation in phospholamban that decreases SERCA2a activity [103], and the same phospholamban mutation was identified in people, albeit with a significantly lower penetrance [104]. Comparing the molecular and functional changes in human and canine patients with this mutation could explain what changes occur in people to limit disease penetrance, or alterations in dogs that allow for more common disease development.

RNA-binding motif protein 20 (RBM20) has a 22 base pair deletion in Giant Schnauzers that is also found in humans [105,106]. This mutation creates improper splicing of genes for titin, Ca^2+^/calmodulin-dependent kinase, and ryanodine receptor 2, which may act as an underlying driver of cardiac arrhythmias [106].

In humans over 900 RBM20 variants have been reported [107], although their connection to DCM remains largely unexplored. In fact, the connection with DCM is tenuous, with a minority of studies that have investigated human cases showing variant co-segregation with DCM [107]. Rodent models created to understand the contribution of RBM20 to DCM have produced mixed results: while RBM20 knockout mice did develop DCM and the disrupted calcium handling changes seen in humans, disease severity was significantly less in rodent models [107]. The early onset and severity of RBM20-associated DCM in Schnauzers represents an opportunity to investigate the role of RBM20 in DCM, using a model that more closely resembles the human condition in terms of both cardiovascular physiology and disease phenotype [108].

#### 4.1.5 Myofilament cross-bridges

The majority of known genes linked to DCM in humans encode for sarcomeric proteins [109] and yet, no studies of canine samples reveal causative mutations in the contractile apparatus. We have shown that cardiac myofilaments isolated from Doberman Pinschers in end-stage DCM exhibit a significant reduction in force development and pronounced impairments in myofilament kinetics [85]. As further evidence of a myofilament component of canine DCM, we also reported that cardiac myosin from Doberman Pinschers with end stage DCM are trapped in a non-sequestered but ATP-conserving superrelaxed state, consistent with an impairment in force development [84]. This finding is in agreement with an earlier study that investigated an E525K myosin mutation linked to human DCM [110], which also found a shift towards the superrelaxed state in disease. While mutations in sarcomeric proteins associated with canine DCM have not been reported, the phenotypic similarities in myofilament dysfunction between human and canine DCM provides strong support for their use as comparative models to identify the molecular basis of DCM in terms of contractile dysfunction, and to test myofilament-targeting therapies like small molecules that promote myosin activation [109].

#### 4.1.6 Extracellular matrix

Extracellular matrix composition and its remodelling by matrix metalloproteinases are important contributors to the development and progression of human DCM [[111], [112], [113]], and is touted as an important area that could be targeted with precision medicine [114]. Doberman Pinschers with DCM are known to exhibit similar changes in extracellular matrix composition alongside changes in metalloproteinase activity [[115], [116], [117], [118]]. The similarities in these extracellular changes allow for knowledge gained by studying canine DCM to be applied to the human condition, including testing of novel therapeutic interventions, and vice versa.

### 4.2 ARVC

In humans, ARVC is a genetic cardiomyopathy driven primarily – but not exclusively – by disruptions in desmosomes [119,120]. Most genes implicated in human ARVC have been excluded as causative mutations for Boxers [121]. However, the 16 different mutations that have been identified in people represent a minority, as >50 % of human cases are idiopathic [46].

In humans, genetic defects in filamin A, dachsous cadherin-related 1, and DAZ interacting zinc finger protein 1, have been identified in people with mitral valve disease, but these mutations are largely limited to a few familial groups [122]. While the pattern of disease development in dogs suggests a polygenic basis, no studies have identified a causative genetic change [123,124]. Lacking a single, definitive genetic mutation as the cause of MMVD, several mechanisms have been proposed that are not necessarily mutually exclusive.

#### 4.2.1 Desmosomal proteins

The only known ARVC mutation in dogs is an 8-base pair deletion in the striatin gene that effectively decreases striatin protein expression [125,126]. Some studies question the causative role of this deletion and suggest that it may simply play a role in disease severity [121]. Others have similarly advanced a multi-gene cause or a role for environmental factors in creating a pathological milieu [46].

While the specific genes involved in ARVC may differ between dogs and humans, both species appear to share a similar disease mechanism. Desmosomal disruptions that impair cell-cell adhesion and increase cardiomyocyte death are key elements in the pathogenesis of ARVC in dogs and people [126]. Using a mouse model of ARVC and combining it with known pathways implicated the human and canine conditions, Montnach and colleagues [127] proposed a common mechanism by which different genetic changes converge in a common disease-causing pathway. In this model the reduction of PKP2 expression in humans or striatin in Boxers decreases the STRIPAK molecular complex, specifically Strip2 expression. The effects on Strip2 lead to calcium dysregulation which underlies increased risk of arrhythmias and cardiac death, and increased Hippo signaling drives the fibrosis and adiposis that are hallmarks of ARVC. Thus, although the specific proteins driving ARVC may differ, the common impact on desmosomes and molecular signaling offers a similar pathological cascade that could shed light on the mechanisms of ARVC, and uncover targets of non-genomic based therapies.

### 4.3 MMVD

MMVD is similarly defined in humans and dogs as the thickening of mitral valve leaflets and disruption of their extracellular matrix, typically resulting in a valvular dysfunction that manifests as regurgitation. Age is a significant factor in both human and canine MMVD as incidence increases in older age groups, even in dog breeds that have an unusually high prevalence of the condition. Canine cases of MMVD are largely homogenous in terms of histopathological changes to the leaflets, while humans develop two distinct subsets of the disease. Barlow's disease, which is marked by mucopolysaccharide deposition and leaflet thickening; elongated or ruptured chordae tendineae; and ultimately mitral valve prolapse, is the form of MMVD that most closely resembles the canine condition [60]. Despite some inter-species differences in MMVD, similarities in key mechanistic elements create a strong potential for comparative investigations that may advance our understanding and treatment of these conditions in both dogs and people.

#### 4.3.1 Serotonin

In both humans and dogs an increased activation of the serotonin pathway has been connected to valvular dysfunction [60]. Serotonin is involved in wound healing and pathological conditions of several organ systems that result in excessive fibrosis [128], a key characteristic of the human condition. The 5-HT_2B_ form of the receptor that has been implicated in mitral valve disease in both humans and dogs, with an increase in the expression of the receptor in diseased samples [129].

There is some evidence that increased serotonin levels occur with MMVD, but these data are variable and offer several mechanisms to promote MMVD. People with neuroendocrine tumors that increase plasma serotonin have higher rates of MMVD [130]. Reduced serotonin transporter activity with selective serotonin-reuptake inhibitors in patients and humans with 5-HTTLPR genotype LL (which is associated with lower serotonin transporter activity) were associated with changes in mitral valve leaflets consistent with MMVD [131].

Increased levels of circulating serotonin were detected in dogs with MMVD [132], and polymorphisms in the serotonin-reuptake transporter that diminish function are connected to disease [133]. Although polymorphisms in the serotonin-reuptake transporter were not found in Cavalier King Charles Spaniels who have the highest rates of canine MMVD [134], circulating serotonin levels are naturally higher in this breed [132,135]. Work from Scruggs and colleagues demonstrated a reduction in the serotonin transmembrane transporter specifically in valve interstitial cells of dogs in late, but not early, stages of MMVD [136]. Further supporting the involvement of a local serotonin pathway as a driver of MMVD, Lacerda et al. found cultured canine mitral valves subjected to strain increased tryptophan hydroxylase 1 expression, along with serotonin levels and markers of activated myofibroblasts [137].

Together these studies support the role of serotonin as a causative player in MMVD in both humans and dogs, with evidence that it may also be involved in the latter stages of disease. The pathways by which the effects of serotonin are enhanced – be it decreased removal, enhanced synthesis, or increased receptor expression – may differ between and within species, but the end result is elevated serotonin signaling in valvular cells in both species.

The pathways of serotonin signaling involved in MMVD are similarly unclear, with several possibilities. Serotonin can activate a mitogenic pathway through an intracellular cascade involving PKC, ERK1/2, and p38 MAPK [123]. The internalization of serotonin and resulting serotonylation of cytoskeletal proteins could also create a MMVD phenotype [123]. The most commonly investigated pathway, however, involves increased activation of the 5-HT_2B_ receptor through increased serotonin levels, overexpression of the receptor, or both, to increase TGF-β expression, which is known to drive fibrosis [123]. The naturally occurring models offered by dogs and the high frequency with which MMVD occurs in several breeds of dog, offers the potential to study disease mechanisms in a model largely analogous to the human condition.

#### 4.3.2 TGF-β

TGF-β induces fibroblast activation and increased fibrosis when the heart is placed under stress [138]. Valvular interstitial cells are induced to take on a phenotype of activated myofibroblasts through TGF-β [138]. Transcriptomic analysis of canine mitral valves revealed changes in genes of the TGF-β pathway [139] and TGF-β signaling is increased in dogs and humans with MMVD [61]. The known effects of TGF-β on inflammation, myofibroblast activation, and extracellular matrix remodelling are consistent with MMVD development, and represent a shared pathway of disease in canine and human patients. This common mechanism allows for the investigation of anti-TGF-β therapies in dogs with MMVD, which may be applicable in humans, and could mediate benefits in the wider group of fibrotic heart conditions.

#### 4.3.3 Microbiome

Although it is not likely the cause of MMVD, disruptions of the microbiome have been implicated in the progression of MMVD into heart failure. The “leaky gut” hypothesis of heart failure proposes a significant role in the movement of gut bacteria into the blood to drive the systemic inflammation that is common in heart failure [140]. In a study of 92 dogs, diversity of gut microbiota decreased in the preclinical stage of MMVD and remained down [141]. While human studies identified a similar relationship between reduced microbiome diversity and heart failure [142], investigations have not been done in the early stages of heart diseases, demonstrating a knowledge gap that exists in this area [141], and identifying information that can be gained from canine patients.

While the gut microbiome has drawn significant research interest for its role as a driver and modifier of cardiovascular diseases, the involvement of the oral microbiome in dogs has generated comparably less attention. This oversight is particularly interesting given the long-standing appreciation of the connection between dental health, oral pathogens, and numerous health conditions, including cardiovascular disease in people [143].

## 5 Diagnostics

### 5.1 DCM

DCM diagnosis in human [144] and veterinary practice [13,38,145] is based on guidelines published by professional societies or practitioners. Human diagnostic protocols include a detailed patient and family history, ECG, laboratory tests (B-type natriuretic peptide (BNP) and troponin), and multimodality cardiovascular imaging (transthoracic echocardiography and cardiac magnetic resonance) [144]. Disagreements among human-focused cardiology societies include the use of computed tomography and nuclear imaging, and ancillary biomarkers such as atrial natriuretic peptide [144]. Screening and the identification of DCM in dogs similarly relies on a patient history, pedigree analysis, physical examination (reduced pulse amplitude, irregular heart rhythms, atrioventricular valve murmur), ECG, echocardiography, and blood tests (BNP and troponin I) [38,145]. For echocardiography, the standards for diagnosing and staging disease are breed-dependent, and biomarker cut-off levels have not been identified for all breeds [145]. In general, the diagnostic tools are similar across human and veterinary medicine, although thresholds for diagnosis differ, including between breeds of dog.

### 5.2 ARVC

There is no single, gold standard test for the diagnosis of ARVC in dogs or people. In humans the diagnosis is based on a series of criteria encompassing ventricular structural-functional abnormalities; ECG disorders; and family history [45]. Diagnostic criteria for dogs are less well-defined and rely heavily on family history or characteristic cardiac arrhythmias in breeds that are pre-disposed to the condition [46].

### 5.3 MMVD

Canine MMVD is diagnosed and staged according to ACVIM consensus guidelines [54], which were adapted from American Heart Association guidelines for human patients [146]. Current guidelines for valvular dysfunction in human patients were written by the American College of Cardiology/American Heart Association Joint Committee on Clinical Practice Guidelines [147]. In both cases echocardiography is the definitive tool for diagnosis, although transesophageal imaging is recommended for humans while transthoracic is the standard for dogs. In some cases, cardiac magnetic resonance may be used with human patients, and radiographs are sometimes used in place of echocardiography for dogs.

#### 5.3.1 β-Adrenergic receptor autoantibodies

Autoantibodies against β-adrenergic receptors are both biomarkers for DCM and mechanistic players in disease development. β-Adrenergic receptor autoantibodies act as receptor agonists and drive a variety of cardiac conditions in humans, including DCM [148,149]. Doberman Pinschers with DCM also have circulating autoantibodies against β-adrenergic receptors, and levels rise with disease severity, presenting a potential opportunity to use these changes as diagnostic and prognostic markers [150]. The recapitulation of the autoantibody profile in dogs with DCM appears to be relatively unique as rodent models of DCM do not have elevated antibody levels that mimic the human condition.

#### 5.3.2 Natriuretic peptides

NT-proBNP is considered a strong biomarker in humans with DCM [151]. A study of 1173 human patients with mild or overt DCM confirmed the reliability of NT-proBNP as a general marker of DCM, but demonstrated that its ability to act as a prognostic biomarker was weak in early stages of the disease [152]. A review of NT-proBNP studies in dogs by Perez and colleagues [153] yielded similar conclusions about the reliability of NT-proBNP as a general biomarker for canine DCM, while pointing out its weakness in early, preclinical stages of the disease.

BNP is a potential marker for humans and dogs with heart failure associated with MMVD. In humans, asymptomatic mitral valve regurgitation is linked to elevated BNP and NT-proBNP levels, making it a suitable marker for early diagnosis [154]. However, the value of BNP as an early marker of MMVD is dogs is unclear, with some studies showing increases only in the late stages of disease [155], whereas others identified changes in the middle [156] or early stages [157]. A similar inconsistency is seen with atrial natriuretic peptide (ANP) levels where some studies report increases only in the late stages in dogs with MMVD [155], while others show an increase that starts early in disease development [157,158]. The ability of ANP to act as a diagnostic or prognostic biomarker of mitral valve disease in humans has not been investigated, opening the possibility that biomarker research in dogs could translate to humans.

Inconsistencies in biomarker use may arise from differences across breeds or different criteria to stage disease, but they may also be the product of using assays designed for human samples. We have previously shown that troponin assays for humans are not always compatible with horse samples, demonstrating the difficulties in applying assays across species [159].

#### 5.3.3 High-sensitivity C-reactive protein (hs-CRP)

Limited studies in dogs with DCM confirm hs-CRP can be a clinically useful marker for DCM, but it is not specific for type of heart failure [160], and no studies have investigated if changes correspond to disease stage. Moreover, hs-CRP rises in dogs following physical activity and its half-life of 19 h has led to caution in interpreting increased CRP levels alone as markers of pathology [161]. The emerging evidence in human patients that hs-CRP may serve as a prognostic biomarker in patients with mild and overt DCM [152] could be further investigated in dogs with a similar pathology, providing more insight into the human application while also testing its effectiveness in a canine population.

#### 5.3.4 Anti-desmoglein-2 antibody

The anti-desmoglein-2 antibody has been identified as a biomarker for the diagnosis of ARVC in human and canine patients. In a study using 65 human and 28 canine samples, Chatterjee and colleagues found antibodies against desmoglein-2 in samples from patients, while healthy controls tested negative [162]. There appeared to be some prognostic value associated with the antibody levels as well. A subsequent prospective study of 46 dogs confirmed the increase in circulating anti-desmoglein-2 antibodies with canine ARVC, but they found similar increases in other cardiac conditions, bringing into question disease specificity of the biomarker [163]. Regardless of disease specificity, this work demonstrates the potential of studying similar cardiac conditions simultaneously to identify common biomarkers.

#### 5.3.5 ECG

P-wave terminal force is an ECG parameter used to identify left atrial enlargement and assess the severity of valvular disease in humans [164,165]. Calderón-Olaguivel and colleagues tested the parameter in 74 dogs with MMVD and found it to be unreliable, as were other P-wave characteristics employed in humans [166]. While these results seem to discount a variety of P-wave parameters suitable for human patients as diagnostic tools for dogs, placement of the ECG leads differs significantly between human and canine patients, and these variations may require an approach tailored more specifically to the unique patterns of the canine ECG [166].

#### 5.3.6 Echocardiography

Echocardiographic parameters found to be effective prognostic markers in humans with MMVD have been validated in dogs. Leaflet-annulus index is a prognostic marker for mitral valve regurgitation in humans [167], with comparable staging benefits for dogs with MMVD [168,169]. Similarly, interventricular inflow time difference, which is used for humans with heart failure [170], is also a good prognostic marker in dogs with MMVD [171]. This example of diagnostic tools developed for humans and modified for canine values demonstrates the truly bidirectional potential of comparative research where both species may realize benefits from work done primarily in the other.

#### 5.3.7 Metabolomics

Despite the known connection between metabolism and heart conditions, there are relatively limited metabolomic investigations done in humans, which could be supplemented by work done in dogs [172]. The metabolic shifts that occur with the development of heart failure offer the opportunity to identify metabolic patterns or “fingerprints”. A 13-year long study that followed 40 dogs from the preclinical, asymptomatic stage of MMVD to heart failure identified lipidomic changes that corresponded to the various stages of MMVD [173]. Changes in 20 lipid species from 7 classes were identified across disease progression, creating a diagnostic pattern for the staging of MMVD. Interestingly, several of the lipid changes were consistent with patterns seen in human heart failure patients, which bodes well for the translatability of these findings. Another study of 57 dogs at various stages of MMVD also found significant changes in serum metabolites, 41 % of which occurred very early in disease development, which both sheds light on the mechanisms of early and often asymptomatic disease, along with identifying potential markers for diagnosis and prognosis [172].

#### 5.3.8 Microbiome

A study of patients with rheumatic heart disease reported significant alterations in the microbial population of the oral cavity, a change that was more pronounced than that seen in the gut microbiome [174]. Furthermore, they found that the β-diversity of the salivary microbiota has strong potential to be used as discriminatory biomarkers for cardiovascular conditions [174].

The oral microbiome of dogs is divergent from humans with markedly different taxa found in each species [175]. However, the general lack of information about the canine oral microbiome [176] limits our understanding of its connection to health conditions. A study of 59,296 dogs with periodontal disease reported a connection between oral bacteria and cardiovascular conditions including endocarditis and cardiomyopathy [177], but a comment in response to this study called into question the methodologies used to make this connection [178]. A more recent review of antimicrobial therapies with periodontal disease highlighted the lack of data connecting dental and cardiovascular health in dogs [179]. Thus, while the human oral microbiome has potential for use in cardiology, in particular as a diagnostic tool, the relative lack of information about the canine oral microbiome serves as an obstacle to translation, and the profound differences between human and canine oral microbiota likely limits the potential for inter-species translation of knowledge.

## 6 Treatments and interventions

### 6.1 DCM

The therapeutic management of DCM in humans is similar to other forms of heart failure [16]. Left ventricular assist devices and cardiac transplantation are recommended for patients in later, end-stages of disease [16]. Medical management includes diuretics and sodium–glucose co-transporter 2 inhibitors (Class I); angiotensin-converting enzyme inhibitors, angiotensin receptor blockers, angiotensin receptor-neprilysin inhibitors, mineralocorticoid receptor antagonists, and β-blockers (Class IIb) [180]. Individual treatment plans may be developed within these guidelines as some studies suggest different degrees of effectiveness depending on the specific aetiology [16]. The medical management of dogs with overt DCM is similar to humans, with recommendations for diuretics and angiotensin converting enzyme inhibitors [181]. Anti-arrhythmics including lidocaine and sotalol are often used despite the lack of evidence that they reduce the risk of sudden cardiac death [181]. One treatment unique to canine cardiology is the first-line use of the positive inotropic agent pimobendan [181]. Generally surgical interventions including left ventricular assist devices and cardiac transplants are not used in veterinary cardiology.

### 6.2 ARVC

Treatment of human ARVC consists of β-blockers as anti-arrhythmic medications or implantable cardioverter defibrillators (ICD) [182]. Dogs with ARVC have similar guideline treatments in the form of β-blockers [46]. The use of ICD in dogs is limited because of issues involving the accurate detection of arrhythmias using instruments designed for humans, and the distress caused by inappropriate discharges [183].

### 6.3 MMVD

In humans, mitral valve regurgitation resultant from MMVD is medically managed with vasodilators, although there is no convincing evidence that this approach reduces the severity of regurgitation [147]. Correction of the valvular issue requires surgical intervention, either in the form of valvular repair (including associated structures) or replacement [39]. Surgical intervention is recommended before the onset of systolic dysfunction [147]. Canine MMVD is medically managed, starting with pimobendan and angiotensin converting enzyme inhibitors when cardiac remodelling is detected (Stage B2) [54]. Dietary treatment in the form of sodium restriction is also recommended. Surgical intervention is indicated at Stage B2, although the opportunity for this intervention is highly limited by cost and access to centres that perform these procedures [54]. Advanced stages of MMVD can be managed medically through a variety of therapies including the continued delivery of pimobendan and angiotensin converting enzyme inhibitors, along with diuretics, nitroprusside, and spironolactone [54], Protocols differ between hospital- and home-based treatments and therapies can be adjusted for severity of symptoms or lack of responsiveness to treatments.

#### 6.3.1 Cardiosphere-derived cells

Work by Hensley and colleagues [184] showed that cardiosphere-derived cells in Doberman Pinschers with DCM were a safe intervention to treat the disease. Subsequent to this preclinical safety study, human-focused clinical trials [185,186] validated the safety and efficacy of cardiosphere-derived cells. The similarities in the pathogenesis of human and canine heart failure were instrumental in constructing a strong foundation for human testing, and support the further use of canine patients with DCM to develop treatments for human patients. Interestingly, in this case the foundation for therapies was laid using human patients with a subsequent translation to dogs. While this direction is relatively rare compared to dog to human translations, it does demonstrate the bidirectional potential of comparative research.

#### 6.3.2 Pimobendan

One interesting area of DCM management for Doberman Pinschers is the highly effective use of pimobendan. Pimobendan is a PDEIII inhibitor and myofilament calcium sensitizer, which reduces cardiac afterload [187] and enhances contractility without an increase in ATP consumption [188]. Human clinical trials revealed significantly higher mortality rates in heart failure patients treated with pimobendan [189]. By contrast, the addition of pimobendan to the standard therapeutic regimen for Doberman Pinschers with DCM significantly prolonged survival [33,190,191] to the point that pimobendan is a standard therapy in canine cardiology [181]. However, pimobendan is not universally effective for canine heart failure as it has proven to be ineffective for the treatment of English Cocker Spaniels [190]. The contrast in the effectiveness of pimobendan between human and canine studies (and even within canine populations) raises an interesting possibility to investigate these discrepant findings through comparative research, and identify specific opportunities to use calcium sensitizers to manage heart failure.

In dogs with MMVD, pimobendan given early in disease development significantly slows progression and delays the onset of heart failure [[192], [193], [194], [195]]. While a body of evidence has been generated to support the use of pimobendan as a guideline therapy [54], similar studies have not tested the effectiveness of this treatment for human patients.

#### 6.3.3 Deoxy-ATP

Another inotropic support proposed as a heart failure therapeutic involves increasing cellular deoxy-ATP levels. The Regnier laboratory has pioneered this approach and demonstrated benefits of a gene-based approach to increase deoxy-ATP levels in cardiac myocytes and treat heart failure [196]. In a proof-of-concept study, we applied deoxy-ATP to cardiac myofilaments from canine DCM patients, and showed that it effectively reduced or eliminated contractile deficiencies that may contribute cardiac dysfunction [85].

#### 6.3.4 β-Adrenergic receptor autoantibodies

The aptamer BC 007 is a 15-mer single-strand DNA oligonucleotide that safely and effectively neutralizes β-adrenergic receptor autoantibodies in Doberman Pinschers with naturally occurring DCM, and prolongs lifespan [197]. These studies using naturally occurring DCM in Doberman Pinschers allowed for the accurate modeling of a human condition to test the safety and effectiveness of a therapeutic intervention, and to identify autoantibodies as diagnostic and prognostic biomarkers. A three-part randomized, double-blind, placebo-controlled study of healthy adults showed BC 007 is safe and well tolerated, with a dose-dependent ability to neutralize β-adrenergic receptor autoantibodies [198]. The virtually simultaneous publication of these studies precluded the use of canine or human data to support use of BC 007 in the complimentary species, but the similar outcomes bolster the suitability of using dogs to model human cardiac conditions for novel therapies.

#### 6.3.5 PKP2 gene therapy

PKP2 variants account for approximately two-thirds of human cases of ARVC, and a mouse model carrying a PKP2 mutation reported in human patients was rescued by an adenovirus vector delivering wildtype PKP2 [199]. Other studies have reported similar results with PKP2 gene therapy and these therapeutics have been approved for Phase I/II clinical trials [182]. While the putative genes causing ARVC differ between dogs and humans, gene therapy may prove valuable for both species, albeit with different gene targets. However, Muller and colleagues noted several genotype-phenotype correlations and have suggested that these connections may require genotype-specific treatments [182], which may limit the applicability of treatments across species.

#### 6.3.6 Surgery

Surgical repair or replacement of diseased valves is increasingly routine in human medicine, to the point that catheter-guided interventions have moved from an option exclusive to high-risk patients, to being utilized more broadly in lower-risk cohorts [200,201]. Canine MMVD poses a unique challenge to using surgical interventions developed for humans. The over-representation of older, small breed dogs with MMVD offers two clinical challenges: low bodyweight and advanced age.

Building off techniques used in humans with mitral valve disease, a surgical process pioneered by Dr. Masami Uechi involving annuloplasty and chordal replacement now allows for surgical repair in the small breed dogs most often afflicted with MMVD [202]. More recently, the transcatheter approach developed for humans has been modified to use a smaller implanted device (“V-clamp”) to repair canine mitral valves, reducing the risks associated with open heart surgery [203,204]. These surgical solutions based on work done with human patients are another example of the bidirectional benefits afforded by the shared knowledge gained from these compatible models of disease.

#### 6.3.7 Diet

While some key differences in myocardial metabolism exist between humans and dogs, such as disparities in GLUT isoform expression, metabolic alterations associated with canine MMVD closely resemble those seen in humans [205]. Metabolic changes have been identified in the early and preclinical stages of disease of dogs, and there is some evidence that nutritional supplements or dietary changes can slow disease progression [206]. Dietary supplementation with omega-3 fatty acids for 1-year reduced volume overload and arrhythmias in dogs with stage B2 and C MMVD [207]. By contrast, a study with similarly staged dogs fed diets enriched in eicosapentaenoic and docosahexaenoic acid showed no meaningful benefits after 6 months [208]. A 6-month, single-blinded, randomized, placebo-controlled dietary intervention study by Li and colleagues [209] tested the effects of a “cardiac protection blend” of nutrients containing medium-chain triglycerides, fish oil, and antioxidants on preclinical MMVD dogs. This dietary blend reduced mitral valve regurgitation and slowed clinical progression of the disease.

In both humans and dogs, ketone body utilization increases in MMVD [172]. These similar metabolic shifts offer the opportunity to test therapeutic ketosis and answer many of the questions concerning its safety and effectiveness [210]. The “cardiac protection blend” of nutrients used by Li and colleagues [209] supplemented ketogenic medium-chain triglycerides [172] showed benefits noted above. In another study, Li and colleagues fed dogs a blend of nutrients that improved fatty acid oxidation, while reducing oxidative stress and inflammation [211]. While there were reductions in metabolic markers of disease, there was no functional assessment, and the long-term benefits of this intervention remain untested.

## 7 Conclusions

Dogs and humans share a number of cardiac conditions that ultimately prove fatal in both species. While traditional rodent models have been highly successful at generating crucial information about several of these pathologies, the sudden induction of a disease-causing stress in an otherwise healthy animal has inherent limitations that serve as obstacles in the translation to human patients. Dogs, with a similar cardiovascular system and predisposition to these pathologies, offer a powerful opportunity to gain insight into each element of disease, from the molecular mechanisms to diagnostics and therapeutics. Similarly, the knowledge of disease development and clinical tools that are used for humans has and may continue to have application in veterinary medicine.

Like any effort to translate knowledge and applications between species, the human-dog bridge also has critical limitations. The common practice of spaying or neutering pets limits the ability to consider the contribution of sex organs to disease or response to treatments. The genetic causes of cardiac conditions are frequently different between the species, which could drive fundamentally different mechanisms of disease and offer unique therapeutic targets. This potential limitation is likely not insurmountable as human heart failure (including genetic cardiomyopathies) has varying and diverse causes, and yet the medical management is largely standardized for the type of heart failure and not the specific aetiology. In fact, even where the underlying causes differ, dogs offer tremendous potential for advancing our understanding of the human condition, and vice versa. By identifying common elements in pathogenesis as well as different agents, critical players in disease may be identified. These core actors could serve as targets for the development of therapies that are more broadly effective at treating cardiac disease in human and canine patients. CRISPR genome editing [212] and stem cell therapies [[213], [214], [215], [216]] have been tested and developed in animal models, including dogs, with the idea of applying these therapies more broadly and irrespective of the specific aetiology. Physiologically, unique mutations that disrupt cardiac function also provide knowledge on how molecules in the heart function, again advancing our fundamental understanding of both veterinary and human physiology. Some elements like the homogeneity of purebred dog breeds are paradoxical, offering the advantage of reducing variability and required sample size to identify significant changes, while at the same time creating the risk that knowledge or clinical advances may not translate to a more diverse community of people.

Comparative research using canine and human samples is not an option for many research programs. The costs of housing dogs throughout disease development is often prohibitively expensive, and the specialized housing for these animals is not routinely found in research facilities, unlike those for traditional animal models like rodents. However, information published in the veterinary medicine literature can be used to provide insight into the aetiology of human conditions by virtue of their pathophysiological similarities. New diagnostic and therapeutic tools may be developed by drawing on veterinary medical reports and directly applying these tools or modifying them to fit human parameters. Schools of veterinary medicine exist throughout the world and researchers should consider collaborations with veterinary researchers and clinicians, as they do human medicine practitioners and investigators. There are certainly administrative, ethical, and legal elements to consider when collecting canine samples for investigation, but in general they are not more cumbersome than those in human medicine.

It is clear that, on balance, dogs and humans can serve as models of heart diseases for each other. Awareness of the strengths and limitations of translation between species should be factored into any comparative research that seeks to extract knowledge from each species, but the same can be said for the use of rodents and other common laboratory animal models for human conditions. Ultimately, the similarities are greater and the differences fewer between humans and dogs than those between rodents and humans, and yet for a number of practical reasons rodents remain the models of choice for biomedical research. These include the high cost of researching using larger animal models like dogs, and ethical challenges faced by veterinarians in using their patients for experimental research – similar to difficulties faced by clinicians in human medicine. The numerous similarities, coupled with the natural occurrence and progression of disease in dogs, offers a tremendous opportunity to investigate common cardiac conditions that are shared by humans and dogs, and advance the health of both.

## CRediT authorship contribution statement

**W. Glen Pyle:** Writing – review & editing, Writing – original draft, Visualization, Funding acquisition, Conceptualization.

## Declaration of Generative AI and AI-assisted technologies in the writing process

AI or AI-assisted technologies were not used for any elements of this manuscript.

## Declaration of competing interest

The authors declare that they have no known competing financial interests or personal relationships that could have appeared to influence the work reported in this paper.
